# The use of multimodal imaging in the evaluation of a patient with central retinal artery occlusion in the setting of asteroid hyalosis: a case report

**DOI:** 10.1186/s13256-021-02774-w

**Published:** 2021-04-02

**Authors:** Timothy M. Janetos, Olga German, Rukhsana Mirza

**Affiliations:** Department of Ophthalmology, Feinberg School of Medicine, Northwestern University, 645 N. Michigan Ave. Suite 440, Chicago, IL 60611 USA

**Keywords:** CRAO, Asteroid hyalosis, Ocular massage, Case report

## Abstract

**Background:**

A central retinal artery occlusion (CRAO) is an ophthalmic emergency due to its strong association with cerebrovascular and cardiovascular morbidity and mortality. A timely diagnosis is necessary but difficult in the setting of dense asteroid hyalosis, as typical fundoscopic findings can be obscured. We present a case where multimodal imaging in an eye with an obscured fundus could lead to timely diagnosis and management of CRAO in a patient with acute vision loss.

**Case presentation:**

A 94-year-old Caucasian woman with a history of exudative macular degeneration presented to the retina clinic with acute vision loss in one eye over the course of an afternoon. The patient had dense asteroid hyalosis, and a direct retinal exam was not possible. Multimodal imaging suggested a CRAO diagnosis. The patient received digital ocular massage directly prior to undergoing fluorescein angiography (FANG), which confirmed the diagnosis. The patient was transported from clinic to the emergency room for an emergency stroke workup, which revealed a spontaneous echo in the left atrial appendage, and the patient was started on antiplatelet therapy. When she presented for follow-up within a week, the patient noted that her vision had improved at the time of digital ocular massage and continued to improve thereafter. Her FANG showed marked reperfusion of the retina, and she subsequently has completely regained her baseline visual acuity.

**Conclusions:**

Multimodal imaging is useful in evaluating visual loss in patients with acute vision loss. In addition, ocular massage is a simple, low-risk intervention that may have benefit in the treatment of acute CRAO. Patients who present to ophthalmologists with an acute CRAO need an emergency referral for evaluation of cerebrovascular and cardiovascular comorbidities.

## Background

Occlusion of the retinal arterial system can encompass transient or permanent impaired blood flow to the inner neurosensory retinal circulation. Central retinal artery occlusions (CRAO) are regarded as an ocular emergency due to their strong association with cerebrovascular and cardiovascular morbidity and mortality [1]. Unlike extra-ocular ischemic cerebrovascular events, there are no validated or well-studied treatment protocols for CRAOs, although a multitude of treatments have been attempted, including conservative therapies such as carbogen inhalation, intravenous acetazolamide, ocular massage, anterior chamber paracentesis, and various other vasodilators [2]. Although none of these therapies has had proven benefit, the physiologic basis for many treatments (i.e. vasodilation to increase retinal blood flow or lowering of intra-ocular pressure to increase perfusion pressure) provides an acceptable option for some patients. More invasive treatments include fibrinolytic therapy, with mixed evidence for use [3, 4].

Most importantly, retinal ischemic events are often the first signs of underlying systemic cardiovascular disease [5]. In a large retrospective population study in Taiwan, the rate of stroke in patients diagnosed with retinal artery occlusions was nearly double that of matched controls (19.61% versus 10.05% over 3 years) [6]. This risk was highest within the first month after diagnosis. A recent meta-analysis of patients with a CRAO showed a 30% rate of acute cerebral ischemia on magnetic resonance imaging (MRI) within 7 days of the diagnosis [7]. Therefore, current recommendations include emergency evaluation with brain and cardiac imaging to risk-stratify and guide treatment aimed at secondary prevention of further cerebrovascular accidents. This makes a timely diagnosis essential. Generally, a CRAO presents with the classic fundus appearance of posterior pole retinal ischemia with a “cherry-red spot,” variable levels of optic disc swelling, and attenuation of the arteries [8]. However, in cases with dense media opacities, such as asteroid hyalosis, this classic appearance is obscured. Asteroid hyalosis is a degeneration of the vitreous causing yellow-white opacities that reflect light and can greatly impede direct examination or color fundus photography. Multimodal imaging including fluorescein angiography (FANG) and optical coherence tomography (OCT) can be effective in bypassing the opacity and used to follow and diagnose retinal disease [9]. We report a case of an individual with dense asteroid hyalosis who presented with a CRAO that was diagnosed in a timely manner and monitored with the use of multimodal imaging.

## Case presentation

A 94-year-old Caucasian woman presented urgently to the retina clinic for changes in her left vision. She was being regularly followed for neovascular age-related macular degeneration in both eyes with OCT-guided management. Her left eye had last received intravitreal anti-vascular endothelial growth factor (anti-VEGF) injection in 2016. Her other ocular history included dense asteroid hyalosis, making direct visualization of the retina difficult. She had a significant prior medical history of hypertension and on presentation had systolic blood pressures in the 200s. She had no relevant family medical history. The day prior to presentation, the patient described a “shadow” over her left vision that had progressed. The following day, she had nearly complete loss of vision in this eye other than a preserved inferior island. She denied headache or jaw claudication. Upon presentation to the clinic, her visual acuity was counting fingers. Confrontational visual fields showed loss throughout all quadrants other than her inferonasal quadrant. She had a left afferent pupillary defect (APD). Her vision in the affected eye was 20/40 at her appointment approximately 1 month earlier.

**Fig. 1:**
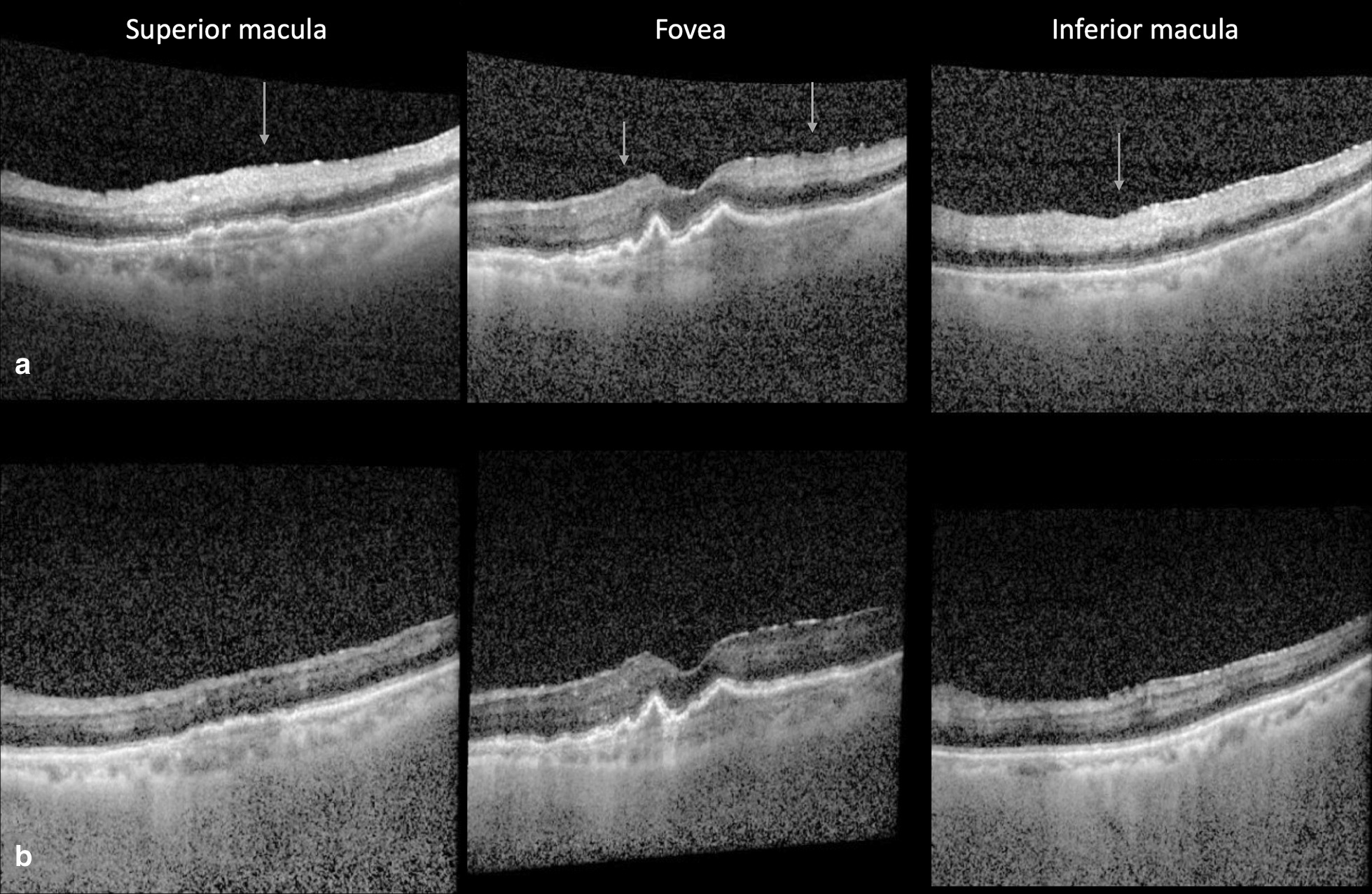
Optical coherence tomography (OCT) of the retina on presentation. OCT imaging of the macula during presentation of acute central retinal artery occlusion (CRAO) **a** showing inner retinal hyperreflectivity and thickening of the macula (arrows) compared to the patient’s previous OCT 1 month earlier (**b**)

**Fig. 2:**
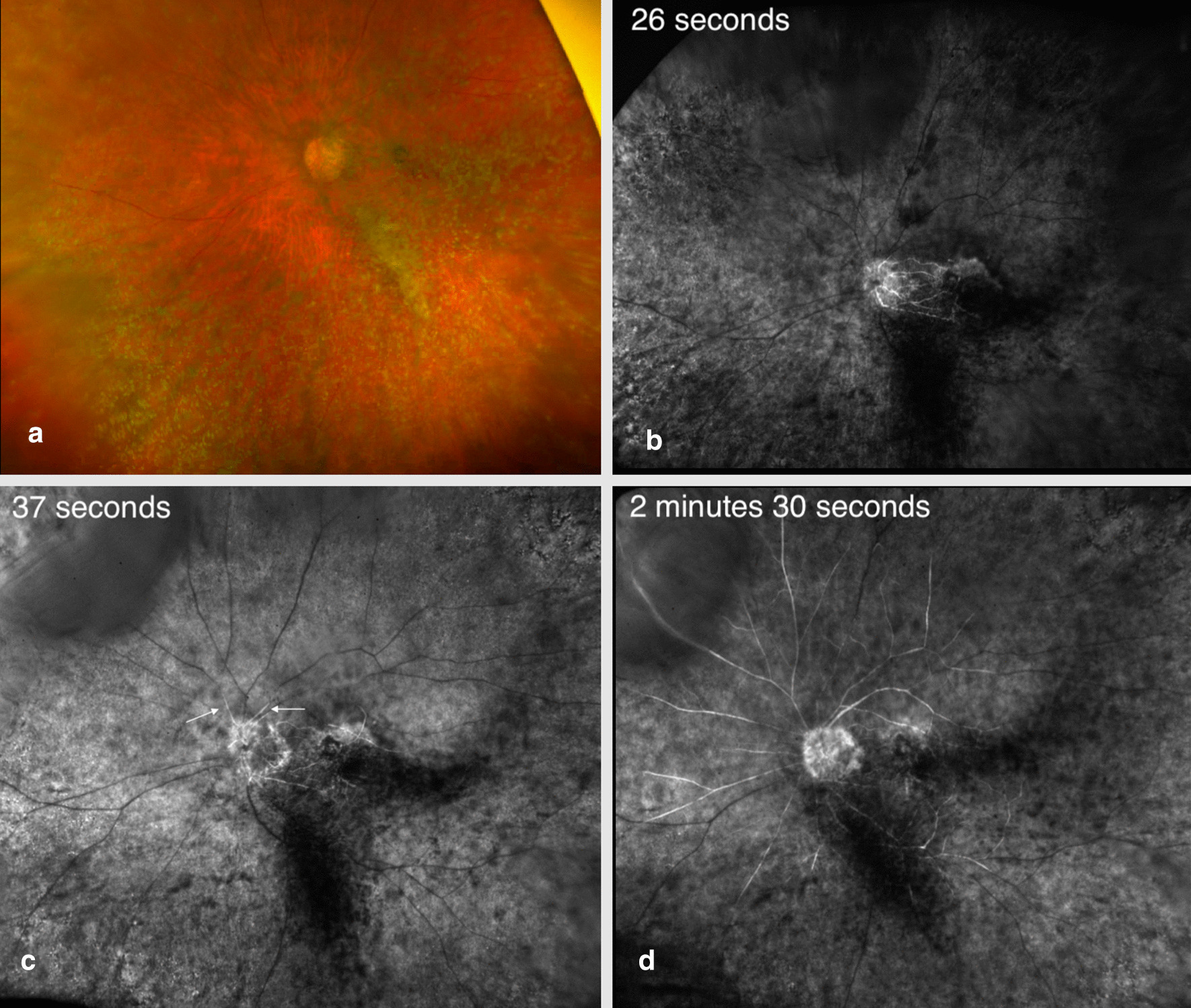
Widefield fluorescein angiography (FANG) on presentation. **a** Widefield fundus photo demonstrates dense asteroid hyalosis, which makes visualization of the macula difficult. There is no cherry-red spot noted. **b** FANG at 26 seconds. There is some flow to the macula. **c** FANG at 37 seconds. There is delayed beginning of arterial phase (arrows). **d** FANG at 2 minutes 30 seconds. There is delayed arterial filling through some of the arterial system

The differential for vision loss was broad and ranged from significant retinal pathology to a neurologic event given the profound vision loss with APD. As it was known that her dilated fundus exam was limited by dense asteroid hyalosis, multimodal imaging was performed to evaluate the patient’s vision loss. During dilation, a B-scan was performed to rule out retinal detachment. After dilation, OCT was obtained, which demonstrated slightly thickened and hyperreflective inner retinal layers that were more prominent superiorly and inferiorly in the macula and likely represented acute ischemia (Fig. 1). At this point, a retinal vascular event was of highest concern, and preparations were made to transfer the patient to the emergency room for urgent stroke workup. A FANG was undertaken to definitively diagnose the cause of vision loss, and ocular massage was performed. Widefield FANG confirmed the diagnosis of a CRAO with some preserved flow in the macula (Fig. 2). Slow arterial flow was first noted at 37 seconds, with patchy arterial flow throughout the peripheral retinal arteries in the late frames. She was subsequently sent directly to the emergency room for imaging and risk stratification. Her giant cell arteritis (GCA) blood workup was negative. Her imaging included MRI and magnetic resonance angiography (MRA) of the head and neck, which were notable for a subacute punctate posterior medial left superior frontal gyrus infarct, thought to likely be embolic. She received a transesophageal echocardiogram, which demonstrated spontaneous echo in the left atrial appendage, a risk factor for thrombosis and subsequent embolus, and was recommended to start apixaban (2.5 mg twice daily) for stroke prevention. However, clopidogrel (75 mg once daily) was started instead due to patient preference.

**Fig. 3:**
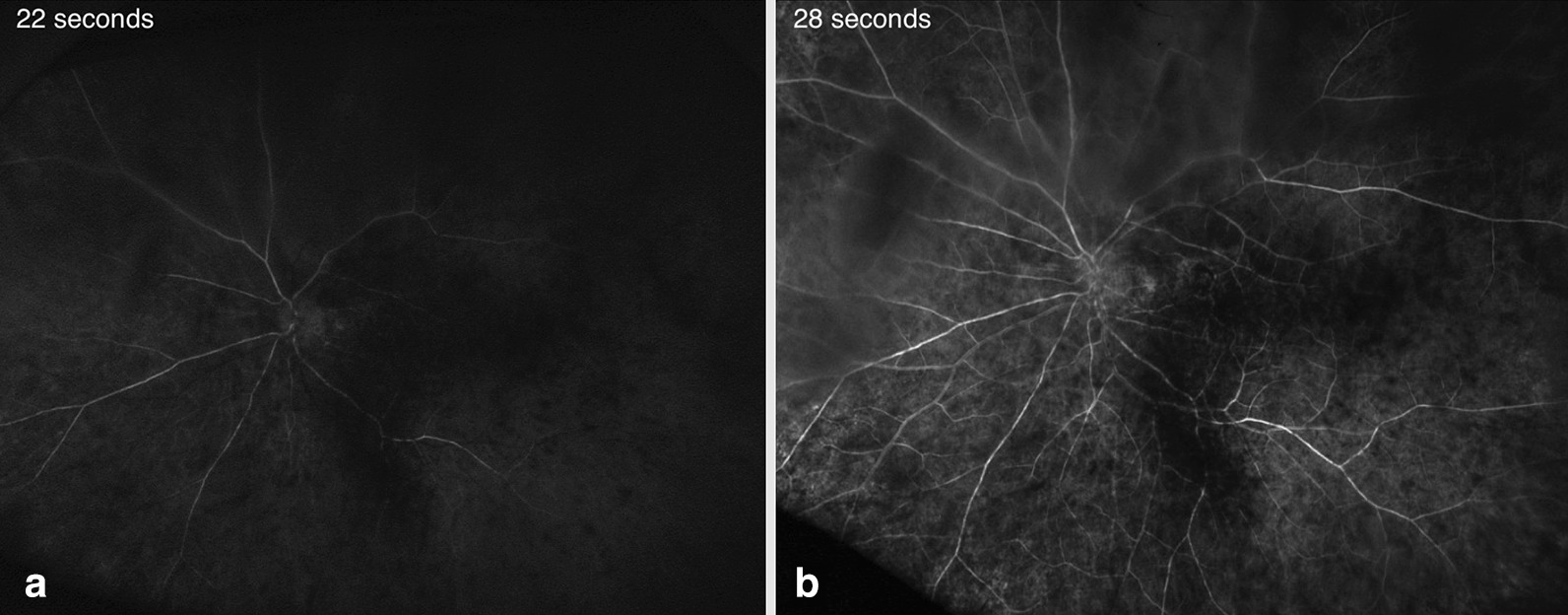
Widefield fluorescein angiography (FANG) on follow-up 4 days after presentation. **a** FANG at 22 seconds. Arterial phase is first seen (of note, the injection time of fluorescein dye was prolonged at 14 seconds). **b** FANG at 28 seconds. Arterial phase is completed.

During follow-up 4 days later, the patient reported that she had experienced gradual improvement of her vision immediately after the digital massage. Best-corrected visual acuity (BCVA) at 4-day follow-up had improved to 20/50−2 in the left eye, along with only slight constriction in her confrontational visual fields. Her repeat FANG now showed markedly improved flow throughout the retina (Fig. 3). Her visual acuity at 10-month follow-up was 20/40−3.

## Discussion

Outcomes after a CRAO are poor, with most patients experiencing profound, permanent visual field loss, and approximately 80% of patients have a final visual acuity of counting fingers or worse [10]. However, visual outcomes are likely dependent on the extent and duration of the occlusion. Some studies estimate that up to 20% of CRAOs have some, even if mild, degree of spontaneous visual improvement. A timely diagnosis and workup are important, as urgent stroke workup needs to be undertaken. A diagnosis can be difficult if the fundus is obscured by media opacity such as dense asteroid hyalosis, and typical fundoscopic findings are not appreciable. We are unaware of any similar reports in the literature illustrating the utility of multimodal imaging for timely diagnosis and monitoring of an eye with CRAO and obscured fundus details. In our case, the diagnosis was made promptly, and the patient was sent to the emergency room without delay. Initial OCT imaging showed hyperreflectivity of the inner retinal layers, which is highly suspicious for a CRAO, as the inner retinal blood supply is derived from the central retinal artery. However, other entities such as paracentral acute middle maculopathy (PAMM) can present similarly on OCT with hyperreflective bands of the middle retina, specifically the inner nuclear layer (INL) due to microvascular capillary ischemia [11]. The definitive diagnosis was made with FANG showing delayed and absent flow through the arterial system. Multimodal imaging has been used previously to diagnosis and manage retinal diseases in the case of a difficult fundus examination. For instance, Motiani *et al*. reported a patient with dense asteroid hyalosis who was diagnosed with a peripheral choroidal melanoma using in part ultra-widefield FANG. This lesion was additionally monitored with imaging modalities as the patient underwent iodine-125 brachytherapy [12].

This case is additionally unique in that our patient fully recovered her vision, with significantly improved arterial flow on follow-up imaging only 4 days after presentation. Hayreh, whose landmark studies on anesthetized monkeys estimated retinal viability time after central retinal artery (CRA) clamping, proposed that visual prognosis is multifactorial and dependent on the duration of ischemia, type and cause of the CRAO, the site of occlusion, and the presence of residual retinal circulation [8]. Schmidt *et al*. proposed a staging system ranging from incomplete to complete based on clinical and FANG findings [13]. An “incomplete” CRAO was defined as diminished visual acuity but with minimal fundus exam findings and delayed but not completely interrupted arterial blood flow. Our patient appeared to develop total visual loss over the course of an afternoon, suggesting a more insidious, rather than complete, occlusion of blood flow.

Our patient also demonstrated initial flow in the macula on FANG, which may have predisposed her to a favorable prognosis. This macular flow may have represented either flow from the posterior ciliary circulation or the initial reperfusion of the CRA, as FANG was taken directly after ocular massage was performed. The utility of ocular massage for treatment of a CRAO remains unknown, and there has been only one prior case study reported on its supposed efficacy [14].

## Conclusions

This case demonstrates that multimodal imaging can be effective in a timely diagnosis of CRAO in a patient with dense asteroid hyalosis. Additionally, although there is minimal evidence for the use of ocular massage, low-risk interventions may have the potential for successful treatment.

## Data Availability

Data sharing is not applicable to this article as no datasets were generated or analyzed during the current study.

## Abbreviations

CRAO: Central retinal artery occlusions
FANG: Fluorescein angiography
OCT: Optical coherence tomography
Anti-VEGF: Anti-vascular endothelial growth factor
APD: Afferent pupillary defect
GCA: Giant cell arteritis
CRA: Central retinal artery
